# Towards a 21st Century Definition of Mental Health – Emerging Trends in Bringing Practice and Research Together

**DOI:** 10.32872/cpe.18657

**Published:** 2025-08-29

**Authors:** Christoph Flückiger, Jan Schürmann-Vengels, Nadine Messerli-Bürgy

**Affiliations:** 1 Clinical Psychology, Department of Psychology, University of Kassel, Kassel, Germany; 2 School of Psychology and Psychotherapy, Witten/Herdecke University, Witten, Germany; 3 Family and Development Research Center, Institute of Psychology, University of Lausanne, Lausanne, Switzerland

Mental health is typically defined in clinical contexts through categorical systems such as the DSM (Diagnostic and Statistical Manual of Mental Disorders) or ICD (International Classification of Diseases). These categorical systems are based on medical standards, where a specific diagnosis is either met or not met (e.g., a Major Depressive Episode is either present or absent). Despite the potential communicative benefits of categorical systems in clinical settings, these classification systems fall short in capturing the complex, multi-faceted nature of mental health (Rief et al., 2023), thereby leading to frustration among practitioners who struggle to reconcile the nuances of human experience with the limitations of existing diagnostic frameworks (e.g., Jensen-Doss & Hawley, 2011). In the context of the rapid advancements in information processing and data sciences, the limitations of existing classification systems are becoming increasingly pronounced. A gap is emerging between the precision in measuring and representing mental health and the above-mentioned, rather crude categorical classification systems for mental disorders. Recent innovations can be illustrated by three developments, among others: The transition from categorical to dimensional, hierarchically structured understanding of mental health severity; the definition of mental health with both individual problems and strengths; and the representation of mental health in a psychosocial and structural context.

## Transition From Categorical to Dimensional, Hierarchically Structured Understanding of Symptom Severity

While a multidimensional understanding of personality disorders has become well established in classification systems, mental health diagnoses with high prevalence rates in the anxiety and depression spectrum continue to be strongly categorical in nature. The HiTOP model presents a counterpoint to this approach and proposes a hierarchical understanding of mental health (Kotov et al., 2021). At a higher level of this hierarchy, internalizing and externalizing behaviors can be defined – a distinction that has been established in the field of child and adolescent psychology for some time (Vergunst et al., 2023). The newer hierarchical understanding of mental health can be illustrated by pathological worrying, for example. In contrast to the DSM and ICD models, which categorize pathological worrying as a primary symptom of generalized anxiety disorder (GAD), the HiTOP model assumes that this symptom may be a key feature of a more general stress factor that crosses traditional diagnostic categories. This view is supported by meta-analytic evidence summarizing the association between worry and mental health based on 138 correlational studies (Vîslă et al., 2022). Notably, the link between worry and mental health was not significantly different between samples of individuals with GAD and those with major depressive disorder, supporting the notion that worry is a common symptom of a broader, generalized distress factor. These findings have important implications for the treatment of worry, suggesting that interventions focusing on worry may be beneficial for a wider range of psychopathologies. This example serves as one of several illustrations demonstrating how a dimensional understanding of mental health can inform the nuanced and sophisticated clinical thinking of practitioners (Hopwood & Sharp, in press).

## Definition of Individual Mental Health Problems and Strength

Mental health is more than merely a response to individual distress. Two-dimensional models of mental health postulate that mental health is more accurately described by two related, but still distinct overall dimensions: Psychological burden (assessed by the degree of psychopathological suffering) and positive mental health, which includes various facets of patient strengths and resources such as subjective life satisfaction, hedonic pleasure, positive affect in everyday life, self-acceptance, personal growth, or finding purpose in life (Schürmann-Vengels et al., 2023). The two affect-related dimensions are not opposites and not strongly related, as two recent meta-analyses demonstrate. Individuals with severe psychological impairments also experience positive states, relatively independent of the severity of their anxiety symptoms (Flückiger et al., 2025) or of depressive moods (Schürmann-Vengels et al., 2025).

Traditional psychotherapies consistently emphasize the importance of strengths and resources as catalysts for therapeutic progress. Moreover, many practitioners acknowledge the limitations of a solely problem-focused understanding of the individuals who come into therapy. A current intersectional task force evaluates capitalization of patient strengths and resources as a 'demonstrably effective' method in terms of therapeutic progress through to treatment termination (Flückiger et al., 2023). This systematic review and meta-analysis examined the effectiveness of strength-based methods in psychotherapy and revealed an association with more favorable immediate outcomes, as well as a small but significant effect on post-treatment outcomes compared to psychotherapy conditions without strength-based personalization. The findings indicate a potential benefit in capitalizing on patient strengths and resources while working on the problems and challenges, specifically by virtue of the systematic and regular assessment of an individual's abilities and motivational readiness, and the corresponding adjustment of mental health interventions. This approach fundamentally redefines the clinical concept of mental health, emphasizing the importance of leveraging an individual's inherent strengths and capacities in the pursuit of psychological well-being.

## Representation of Mental Health in Psychosocial and Structural Contexts

Mental health encompasses more than the symptoms and abilities of an individual person. Mental suffering is caused and experienced in and with others. Mental health issues develop and are maintained within a social context where factors like relationship dynamics (e.g. within families, peers, at work or school) and chronic social stress exposure (ex. conflicts, social exclusion, discrimination, low socioeconomic status) negatively influence an individual’s well-being. Severe stress exposure of family members such as critical life events (e.g. the birth of a child or physical health issues of a family member) can not only increase the risk of mental health issues in individuals but also in their partners and children.

In turn, developed mental health issues can negatively impact an individual’s social environment by straining relationships, perpetuating stigmatization and/or provoking social exclusion. As another example, numerous studies have shown that marginalized groups experience disproportionate levels of mental distress due to additional stressors, such as discrimination, arising both from their immediate social environments and from broader societal structures (Hatzenbuehler et al., 2024). These examples demonstrate that the definition of mental health should not just be reduced to individual-level factors and instead reflect a complex psychosocial phenomenon that needs to be captured in future mental health assessments (Chronister et al., 2021).

The above-mentioned three trends highlight the need for a reciprocal exchange of knowledge between research and practice to define mental health, where practice is informed by current research, and research, in turn, is grounded in the everyday realities and challenges of clinical practice. These developments, however, also provide compelling evidence that the convergence of research and practice is considerably advancing.
